# Dynamic soliton solutions and stability analysis of the (2+1)-dimensional Wazwaz Kaur Boussinesq equation using an efficient method

**DOI:** 10.1038/s41598-025-28602-5

**Published:** 2026-02-11

**Authors:** Nivan M. Elsonbaty, Hamdy M. Ahmed, Niveen M. Badra, Wafaa B. Rabie

**Affiliations:** 1 Basic Sciences Department, The British University in Egypt, Cairo, Egypt; 2 Department of Physics and Mathematics Engineering, Faculty of Engineering, Ain Shams University, Cairo, Egypt; 3 Department of Physics and Engineering Mathematics, El-Shorouk Academy, Higher Institute of Engineering, El-Shorouk City, Cairo Egypt; 4 Department of Mathematics, Faculty of Science Luxor University, Taiba, Luxor Egypt

**Keywords:** (2+1)-dimensional Wazwaz Kaur Boussinesq equation, Analytic method, Soliton solutions, Stability analysis, nonlinear dynamics., Mathematics and computing, Optics and photonics, Physics

## Abstract

This paper presents the first application of the Modified Extended Direct Algebraic (MEDA) method to the (2+1)-dimensional Wazwaz-Kaur-Boussinesq equation, a model governing wave dynamics in shallow waters. The approach successfully uncovers previously unreported classes of exact solutions, including combo dark–singular solitons and Jacobi elliptic function solutions. The spectrum of obtained solutions–which also encompasses bright, dark, and singular solitons, as well as hyperbolic, periodic, exponential, and rational functions–reveals rich and complex soliton dynamics. A comprehensive stability analysis confirms the robustness of these solutions under perturbation. These results significantly advance the understanding of wave propagation in nonlinear systems, providing valuable insights for applications in fluid dynamics, nonlinear optics, and plasma physics, while demonstrating the efficacy of the MEDA method for tackling complex nonlinear evolution equations.

## Introduction

In certain physical systems, wave propagation is governed by the Wazwaz-Kaur-Boussinesq equation, a nonlinear partial differential equation with two spatial dimensions and one temporal dimension. This equation is an extension of the Boussinesq equation, originally used to describe shallow water wave behavior and related phenomena^1,2^. It represents the development of waves, or solitons, in a two-dimensional spatial and one-dimensional temporal framework^3^. The nonlinear nature of this equation facilitates the study of complex wave interactions^4^, propagation dynamics, and soliton formation in multidimensional spaces. The principal impetus for this investigation derives from the substantial applicability of the (2+1)-dimensional Wazwaz-Kaur-Boussinesq equation across pivotal domains of physical sciences and engineering. Serving as a significant extension of the classical Boussinesq paradigm, this equation furnishes a more realistic mathematical framework for simulating complex wave phenomena exhibiting inherent two-dimensional characteristics. The model finds paramount utility in multiple application domains shallow water wave dynamics, where it facilitates accurate modeling of surface wave propagation with coefficients $\mu _1$ and $\mu _2$ correlating to physical parameters including vertical fluid displacement and characteristic wave celerity, proving indispensable for coastal engineering applications and tsunami prediction methodologies, nonlinear optics and plasma physics, enabling comprehensive description of optical soliton propagation in bulk media alongside wave dynamics in magnetized plasma environments; and generalized nonlinear wave theory, where it serves as a fundamental prototype for investigating formation mechanisms and stability properties of sophisticated wave structures in elevated dimensional contexts. Through rigorous examination of this model, our research aims to formulate novel analytical solutions that can function as benchmark references for numerical validation while simultaneously advancing theoretical comprehension of wave stability in these practical scenarios. The relentless quest for exact solutions to nonlinear partial differential equations (NLPDEs) has catalyzed the development of numerous sophisticated analytical methodologies, continuously expanding the arsenal available to researchers. Contemporary scholarly advances have demonstrated remarkable efficacy of both innovative and refined techniques in extracting soliton solutions and deciphering complex nonlinear dynamics. Exemplary contributions include the successful implementation of the unified auxiliary equation method for the complex Ginzburg-Landau model^5^, alongside the generalized exponential rational function approach that has yielded novel soliton solutions for the nonlinear Schrödinger equation^6^. Subsequent investigations have elucidated soliton dynamics in magneto-optic waveguides through the novel Kudryashov framework^7^, while the modified Khater methodology has been employed to analyze intricate resonant nonlinear wave patterns^8^. Furthermore, rigorous examination of the stochastic Radhakrishnan-Kundu-Lakshmanan model has illuminated the profound impact of stochastic fluctuations on soliton propagation characteristics^9^. Recent groundbreaking studies have significantly advanced this field through innovative applications of fractional calculus and dynamical systems theory. Notable contributions include: the exploration of Gross-Pitaevskii model in Bose-Einstein condensates revealing novel solitary wave features and dynamical behavior^10^; comprehensive analysis of multistability and sensitivity in fractional Sharma-Tasso-Olver equation^11^; investigation of optical wave structures in fractional nonlinear integrable coupled Kuralay equation^12^; examination of chaotic structures and sensitivity in nonlinear fractional longitudinal wave equation^13^; and detailed study of solitary wave dynamics in ultrasound imaging applications^14^. These methodological advancements, among others, underscore the enduring vitality of analytical approaches in resolving the intricacies of nonlinear systems, providing profound physical insights often unattainable through exclusively numerical computations. The modified extended direct algebraic (MEDA) methodology employed in this research confers several distinctive advantages compared to conventional analytical techniques. It facilitates systematic generation of an extensive spectrum of exact solutions–encompassing bright, dark, singular, and composite soliton structures–within a unified mathematical framework. The method exhibits exceptional computational efficiency through reduction of the complex partial differential equation to a manageable ordinary differential equation system, enabling streamlined parameter determination via algebraic computations. Its methodical implementation ensures reproducible results across diverse nonlinear evolution equations, eliminating prerequisite assumptions regarding solution forms while generating robust outcomes through an auxiliary equation mechanism. Most significantly, the methodology yields physically pertinent solutions that effectively bridge mathematical constructs with observable phenomena in optical systems, plasma environments, and fluid dynamic applications. Solutions to this equation are commonly utilized in fields such as nonlinear physics, fluid dynamics, and the mathematical modeling of various physical systems, aiding in the understanding of nonlinear wave behavior in multidimensional contexts and the mathematical description of $(2+1)$ dimensions. In addition to advancing the general theory of differential equations, the study of emerging equations aids in the advancement of pertinent analytical and numerical techniques. The precise solution of the nonlinear equation about solitary waves provides a strong and analytical comprehension of the associated physical system, particularly in regions where numerical solutions are ineffective. The last several decades have seen a widespread equipping of contemporary theories with effective techniques and trustworthy algorithms to tackle the difficult riddles of intricate natural occurrences, besides to their practical uses, the close-form solutions of nonlinear partial differential equations (NLPDEs) aid numerical solvers in stability analysis and allow them to compare the accuracy of their solutions (see^10–32^). The following is the formula for the (2+1)–dimensional WKBE describing the flow in a shallow inviscid layer, viewed as^33,34^:

1 $$\begin{aligned} P_{\text {tt}} \,-P_{\text {xx}}\,-\,\mu _1 (P_{\text {xx}})^2{}\,-\,\mu _2 P_{\text {xxxx}}\,+\mu _3 P_{\text {ty}}\,+\,\frac{ \mu _3^2}{4}\, P_{\text {yy}}=0, \end{aligned}$$

where, $P=P( x, y, t )$ are real-valued functions that denote the wave envelop, whose coefficients ($\mu _i; \, i= 1, 2, 3$) are real constants, and whose coefficients $\mu _1$ and $\mu _2$ are determined, respectively, by the vertical extent of the fluid and the characteristic speed of the elongated wave profiles in shallow water.

The Wazwaz-Kaur-Boussinesq equation (WKBE) represents a significant advancement in the theoretical modeling of nonlinear wave phenomena. Historically, the classical Boussinesq equation dates back to the 19th century when Joseph Boussinesq derived it to describe solitary waves in shallow water channels. Over the decades, various extensions have been developed to enhance its applicability. The particular (2+1)-dimensional form given in Eq. (1) was introduced more recently by Wazwaz and Kaur to overcome the limitations of one-dimensional models by incorporating an additional spatial dimension, thereby enabling the study of more complex wave interactions and pattern formations. It is worth noting that Eq. (1) represents a recent generalization of the classical Boussinesq framework, introduced by Wazwaz and Kaur, which extends the model into a (2+1)-dimensional setting. This generalization enhances the capacity of the equation to capture complex multidimensional wave phenomena, thereby establishing its originality and relevance in current research. The physical applications of this model are substantial and diverse. Primarily, it describes wave propagation in shallow inviscid fluids, making it directly applicable to tsunami modeling, coastal engineering, and the study of rogue waves in oceanography. Beyond hydrodynamics, the equation also finds relevance in nonlinear optics for modeling light propagation in specific media, and in plasma physics for describing certain wave phenomena. The parameters $\mu _i$ carry distinct physical meanings: $\mu _1$ and $\mu _2$ relate to nonlinearity and dispersion effects from fluid properties, while $\mu _3$ introduces coupling between temporal and spatial evolution, enabling the description of more complex wave dynamics. This study presents the first application of the Modified Extended Direct Algebraic (MEDA) method to the (2+1)-dimensional Wazwaz-Kaur-Boussinesq equation, yielding novel solution classes including combo dark–singular solitons and Jacobi elliptic function solutions previously unreported in literature. Diverse exact solutions–bright, dark, singular solitons, hyperbolic, periodic, exponential, and rational functions–are obtained, revealing complex soliton dynamics. Stability analysis confirms solution robustness, providing valuable insights for fluid dynamics, nonlinear optics, and plasma physics applications. Additionally, contour, 2D, and 3D plots illustrate the model’s physical behaviors.

The following sections provide the study roadmap: Sect. 2 briefly describes the direct algebraic technique used. Section 3 models the (2 + 1) dimension WKBE (1). Section 4 offers a thorough stability analysis of the (2+1)-dimensional WKBE. Section 5 provides 2D and 3D graphical representations of selected solutions to illustrate their physical characteristics, and Sect. 6 summarizes the results of this study.

## The proposed technique

These publications^35–38^give an overview of the MEDA method. Taking into consideration the nonlinear partial differential equation (NLPDE) below:

2 $$\begin{aligned} Q \left( \,P,~ \frac{\partial P}{\partial t},~ \frac{\partial P}{\partial x}~,\frac{\partial P}{\partial y}~, \frac{\partial ^2 P}{\partial x^2}~,\frac{\partial ^2 P}{\partial y^2}~,\frac{\partial ^2 P}{\partial t^2}~,\frac{\partial P}{\partial x \partial t}~, ...\,\right) = 0, \end{aligned}$$

where $\mathcal {Q}$ is a polynomial of $P (x,\,y,\, t)$ and its partial derivatives for time *t* and space $(x,\,y)$. At the moment, the following are the main stages of the proposed methodology:

*Step* I: Using the ensuing transformation:

3 $$\begin{aligned} P~(x,~y,~t)\,=\,A \,(\xi ) , \qquad \qquad \xi =\alpha _1\,x +\alpha _2\,y-\,\eta t,\qquad \eta \ne 0, \end{aligned}$$

where the wave numbers and soliton frequency are shown by $\alpha _1\,$, $\alpha _2$, and $\eta$.

*Step* II: Thus, Eq. (2) becomes:

4 $$\begin{aligned} Q (A,\, \frac{d A}{d \xi }, \, \frac{d^2 A}{d \xi ^2}, \,\frac{d^3 A}{d \xi ^3}, \,\frac{d^4 A}{d \xi ^4},\frac{d^5 A}{d \xi ^5},\ldots \ldots ) = 0. \end{aligned}$$

The fundamental idea behind the approach is that the solution to Eq. (4) may be represented as follows:

5 $$\begin{aligned} A(\xi )=\sum _{i=-N}^{N} \,\gamma _i~ \mathcal {R}^i(\xi ). \end{aligned}$$

The following differential equation has an explicit solution provided by $\mathcal {R}\,(\xi )$ and $\gamma ^2_N + \gamma ^2_{-N} \ne 0$, where $\gamma _i$ represents real-valued constants that must be found:

6 $$\begin{aligned} \mathcal {R}'(\xi )= \sqrt{\tau _0+\tau _1 ~\mathcal {R}(\xi )+\tau _2 ~\mathcal {R}^2(\xi )+\tau _3 ~\mathcal {R}^3(\xi )+\tau _4~ \mathcal {R}^4(\xi )+\tau _6 ~\mathcal {R}^6(\xi )}, \end{aligned}$$

where the constant real numbers  $\tau _n,~(n=0,1,2,3,4,6)$ depend on the possible outcomes of the employed approach.

*Step* III: We apply the balancing principle to get the integer *N*.

*Step* IV: Keeping in mind Eq. (6), one may construct a polynomial of $\mathcal {R}(\xi )$ by substituting Eq. (5) in Eq. (4). One can equal the coefficients of $~\mathcal {R}(\xi )^{i}$ to zero, which yields an algebraic equation system and, consequently, a range of solutions for solitary waves.

*Step* V: Several types of exact solutions could be obtained to equation (6) as follows:

**Case  1:** When  $\tau _0=\tau _1=\tau _3=\tau _6=0,$ then:

$$\begin{aligned} A_{1.1} (\xi )=\sqrt{-\frac{\tau _2}{\tau _4}}\,\text {sech}\left[ \xi \,\sqrt{\tau _2} \right] ,\,\tau _2>0~\text {and}~\tau _4<0, \end{aligned}$$

$$\begin{aligned} A_{1.2}(\xi )=\sqrt{-\frac{\tau _2}{\tau _4}}\,\sec \left[ \xi \, \sqrt{ -\tau _2} \right] ,\; \tau _2<0~\text {and}~\tau _4>0. \end{aligned}$$

**Case 2:** If  $\tau _0=\frac{\tau _2^2}{4 \tau _4},~\tau _1=\tau _3=\tau _6=0,$ then:

$$\begin{aligned} A_{2.1} (\xi )=\sqrt{-\frac{\tau _2}{2 \tau _4}} \,\tanh \left[ \xi \, \sqrt{-\frac{\tau _2}{2}}\right] ,\;\tau _2<0~\text {and}~\tau _4>0, \end{aligned}$$

$$\begin{aligned} A_{2.2} (\xi )=\sqrt{\frac{\tau _2}{2 \tau _4}} \,\tan \left[ \xi \,\sqrt{\frac{\tau _2}{2}}\right] ,\,\tau _2>0~\text {and}~\tau _4>0. \end{aligned}$$

**Case 3:** If  $\tau _3=\tau _4=\tau _6=0,$ then:

$$\begin{aligned} A_{3.1} (\xi )=\frac{\tau _1 }{2 \tau _2}\,\left[ \sinh \left[ 2 \xi \, \sqrt{\tau _2} \right] -1\right] ,\,\tau _2>0~\text {and}~\tau _0=0, \end{aligned}$$

$$\begin{aligned} A_{3.2} (\xi )=\frac{\tau _1 }{2 \tau _2}\left[ \sin \left[ 2 \,\xi \,\sqrt{-\tau _2} \right] -1\right] , \,\tau _2<0~\text {and}~\tau _0=0, \end{aligned}$$

$$\begin{aligned} A_{3.3} (\xi )=\exp ^{ (\xi \, \sqrt{\tau _2})}-\frac{\tau _1}{2 \tau _2},\, \,\tau _0=\frac{\tau _1^2}{4 \tau _2}~\text {and}~\tau _2>0. \end{aligned}$$

**Case 4:** If  $\tau _0=\tau _1=\tau _2=\tau _6=0,$ then:

$$\begin{aligned} A_{4.1} (\xi )=\frac{4 \tau _3}{ \tau _3^2\,\xi ^2-4 \tau _4}. \end{aligned}$$

**Case 5:** If  $\tau _0=\tau _1=\tau _6=0,$ then:

$$\begin{aligned} A_{5.1} (\xi )=-\frac{\tau _2}{\tau _3}\,\left[ \tanh \left[ \frac{\xi }{2} \, \sqrt{\tau _2} \right] +1\right] , \,\tau _2>0,~\text {and}~\tau _2= \frac{4\tau _4}{\tau ^2_3}, \end{aligned}$$

$$\begin{aligned} A_{5.2} (\xi )=-\frac{\tau _2}{\tau _3} \, \left[ \coth \left[ \frac{ \xi \, }{2}\,\sqrt{\tau _2}\right] +1\right] ,\,\tau _2>0,~\text {and}~\tau _2= \frac{4\tau _4}{\tau ^2_3}, \end{aligned}$$

$$\begin{aligned} A_{5.3} (\xi )=\frac{\tau _2 \, \text {sech}^2\left[ \frac{ \xi \,\sqrt{\tau _2}}{2}\right] }{2 \,\sqrt{\tau _2 \tau _4} \tanh \left[ \frac{ \xi \, \sqrt{\tau _2}}{2}\right] -\tau _3},\, \tau _3^2\ne 4 \tau _2\, \tau _4,~\tau _2>0, \, \text {and}~\tau _4>0, \end{aligned}$$

$$\begin{aligned} A_{5.4} (\xi )=\frac{\tau _2\, \sec ^2\left[ \frac{ \xi \, \sqrt{ -\tau _2}}{2}\right] }{2 \,\sqrt{ -\tau _2 \tau _4} \tan \left[ \frac{ \xi \, \sqrt{ -\tau _2}}{2}\right] +\tau _3},\, \tau _3^2\ne 4 \tau _2\,\tau _4,~\tau _2<0, \, \text {and}\,\tau _4>0. \end{aligned}$$

**Case 6:** If  $\tau _2=~\tau _4=~\tau _6=0,$ then:

$$\begin{aligned} A_{6.1}(\xi )=\wp \left( \frac{ \xi \, \sqrt{\tau _3}}{2};-\frac{4 \tau _1}{\tau _3},-\frac{4 \tau _0}{\tau _3}\right) ,~\tau _3>0. \end{aligned}$$

**Case 7:** If  $\tau _1=~\tau _3=0,$ then:

$$\begin{aligned} A_{7.1} (\xi )=\sqrt{\frac{2 \tau _2 \text {sech}^2\left( \xi \, \sqrt{\tau _2}\right) }{2 \sqrt{\tau _4^2-4 \tau _2~ \tau _6}-\left( \sqrt{\tau _4^2-4 \tau _2 \tau _6}+\tau _4\right) \text {sech}^2\left[ \xi \, \sqrt{\tau _2}\right] }},~\tau _2>0, \end{aligned}$$,

$$\begin{aligned} A_{7.2} (\xi )=\sqrt{\frac{2 \tau _2\sec ^2\left[ \xi \, \sqrt{ -\tau _2}\right] }{2 \sqrt{\tau _4^2-4 \tau _2 s_6}-\left( \sqrt{\tau _4^2-4 \tau _2 \tau _6}-\tau _4\right) \sec ^2\left[ \xi \, \sqrt{ -\tau _2}\right] }},~\tau _2<0, \end{aligned}$$,

$$\begin{aligned} A_{7.3} (\xi )=\sqrt{\frac{8 \tau _2 \tanh ^2\left[ \xi \, \sqrt{-\frac{\tau _2}{3}}\right] }{3 \tau _4 \left( \tanh ^2\left[ \xi \,\sqrt{-\frac{\tau _2}{3}} \right] +3\right) }},~\tau _2<0, \end{aligned}$$,

$$\begin{aligned} A_{7.4} (\xi )=\sqrt{\frac{8 \tau _2 \tan ^2\left[ \xi \, \sqrt{\frac{\tau _2}{3}}\right] }{3 \tau _4 \left( 3 - \tan ^2\left[ \xi \, \sqrt{\frac{\tau _2}{3}}~ \right] \right) }},~\tau _2>0. \end{aligned}$$.

**Case 8:** If  $\tau _1=\tau _3=\tau _6=0,$ then:

**Table:** 

*No*.	$\tau _0$	$\tau _2$	$\tau _4$	$A(\,\xi \,)$
1	1	$-(n^2+1)$	$n^2$	$\text {sn}\,(\xi ,\, n)~\text {or}~\text {cd}\,(\xi ,\, n)$
2	$n^2\,-1$	$-(n^2\,-2)$	-1	$\text {dn}\,(\xi ,\, n)$
3	$-\,n^2$	$2n^2-1$	$1-\,n^2$	$\text {nc}\,(\xi ,\, n)$
4	- 1	$2-\,n^2$	$n^2\,-1$	$\text {nd}\,(\xi ,n)$
5	1	$2-4n^2$	1	$\text {dn}(\xi ,\, n)\; \text {nc}(\xi ,\, n)\; \text {sn}(\xi ,\, n)$
6	$n^4-2n^3+n^2$	$-\frac{4}{n}$	$-n^2+6n-1$	$\frac{\tau ~\text {cn}(\xi ,\, n) ~\text {dn}(\xi , n)}{\,1+\,\tau ~\text {sn}^2(\xi , n)}$
7	$\frac{1}{4}$	$\frac{n^2}{2}-1$	$\frac{n^4}{4}$	$\frac{\text {sn}(\xi ,\, n)}{\,1 + \text {dn}(\xi ,\, n)\,}~\text {or}~\frac{\text {cn}(\xi , \,n)}{~\sqrt{1-\tau ^2}~+~\text {dn}(\xi ,\, n)~}$

After that, several precise wave solutions to the Eq. (1) may be obtained by putting the found constants $\gamma _i$, into Eq. (5) together with the general solutions of Eq. (6).

## Retrieval exact wave solutions

Using travelling wave solution defined in Eq. (3) into Eq. (1) to obtain:

7 $$\begin{aligned} \alpha _1^4 \,\mu _2 \,A^{(4)}\,-\,\left( \frac{1}{4} \left( \alpha _2 \, \mu _3\,-\,2 \,\eta \right) {}^2\,-\,\alpha _1^2\right) \,A''\,+\,2\,\mu _1\, \alpha _1^2 \,A \, A''\,+\,2 \alpha _1^2\, \mu _1 \,A'^2=0. \end{aligned}$$

It is worth noting that Eq. (7), written as an ODE, can be further integrated once to obtain a simplified form. This integration step facilitates the verification of the homogeneous balance number *N*, ensuring consistency of the solution structure before applying the MEDA expansion.

According to the strategy described in Step II of the previous section, the general solution to Eq. (7) can be expressed as:

8 $$\begin{aligned} A(\xi )=\gamma _0 + \gamma _1 \mathcal {R}(\xi ) + \gamma _2 \mathcal {R}^2(\xi ) + \frac{\gamma _{-1}}{\mathcal {R}(\xi )} + \frac{\gamma _{-2}}{\mathcal {R}^2(\xi )}. \end{aligned}$$

Subsequently, Eq. (8) is substituted into Eq. (7), while employing the auxiliary relation given in Eq. (6). By equating the coefficients of different powers of $\mathcal {R}(\xi )$ to zero, a nonlinear algebraic system for the unknown constants $\gamma _i$ is obtained, from which the exact soliton solutions are derived.

By substituting Eq. (8) and Eq. (6) from Sect. 2 into Eq. (7) and setting the coefficients of $\mathcal {R}^i(\xi )$ to zero, the parameters $\gamma _0$, $\gamma _1$, $\gamma _{-1}$, $\gamma _2$, and $\gamma _{-2}$ are determined. This process leads to a nonlinear system of equations (NLAEs), which can be solved using Mathematica to compute the values of these parameters. The subsequent results are as follows:

**Case** A: Upon  $\tau _0=\tau _1=\tau _3=\tau _6=0$, the following outcomes are produced:

$$\begin{aligned} \gamma _1=~\gamma _{-1}=~\gamma _{-2}=~0,~\gamma _2=-\frac{6 \alpha _1^2 \mu _2 \tau _4}{\mu _1} ,~\text {and}~ \gamma _0=\frac{4 \left( -\alpha _2 \eta \mu _3-\alpha _1^2+\eta ^2\right) -16 \alpha _1^4 \mu _2 \tau _2+\alpha _2^2 \mu _3^2}{8 \alpha _1^2 \mu _1}. \end{aligned}$$

From the above set, the following solutions are obtained under condition $\alpha _1^2\, \mu _1\ne 0$:

The bright soliton with $\tau _2>0$, and the singular periodic solutions with $\tau _2<0$ are described below:

9 $$\begin{aligned} \begin{aligned}&P_{1.1,1}(\,x,\,y,\,t)=\frac{\left( \alpha _2 \mu _3-2 \eta \right) {}^2-4 \alpha _1^2-16 \alpha _1^4 \mu _2 \tau _2 \left( 1-3 \text {sech}^2\left( \left( \alpha _1 x\,+\,\alpha _2 y\,-\eta t\right) \right) \sqrt{\tau _2}\right) }{8 \alpha _1^2 \mu _1},\\ \end{aligned} \end{aligned}$$

10 $$\begin{aligned} \begin{aligned}&P_{1.1,2}( \,x, y, t )=\frac{\frac{1}{4} \left( \alpha _2 \mu _3-2 \eta \right) {}^2-\alpha _1^2-4 \alpha _1^4 \mu _2 \tau _2 \left( 1-3 \sec ^2\left( \left( \alpha _1 x+\alpha _2 y-\eta t\right) \sqrt{-\tau _2}\right) \right) }{2 \alpha _1^2 \mu _1},\\ \end{aligned} \end{aligned}$$

11 $$\begin{aligned} \begin{aligned}&P_{1.1,3}( \,x, y, t )=\frac{\frac{1}{4} \left( \alpha _2 \mu _3-2 \eta \right) {}^2-\alpha _1^2-4 \alpha _1^4 \mu _2 \tau _2 \left( 1-3 \csc ^2\left( \left( \alpha _1 x+\alpha _2 y-\eta t\right) \right) \sqrt{-\tau _2}\right) }{2 \alpha _1^2 \mu _1}.\\ \end{aligned} \end{aligned}$$

**Case** B: Upon  $\tau _1=\tau _3=\tau _6=0, \, \text {and}\,\tau _0=\frac{\tau _2^2}{4 \tau _4}$, the following outcomes are produced:

$$\begin{aligned} \begin{aligned}&\texttt {(2.1)}~\gamma _1~=\gamma _{-1}~=\gamma _2~=0,~\gamma _{-2}=-\frac{3 \alpha _1^2 ~\mu _2~ \tau _2^2}{2 \mu _1~ \tau _4} ,~ \text {and}~\gamma _0=\frac{4 \left( -\alpha _2 ~\eta ~ \mu _3-4 \alpha _1^4 \mu _2 \tau _2-\alpha _1^2+\eta ^2\right) +~\alpha _2^2 ~\mu _3^2}{8 \alpha _1^2 \mu _1}.\\ \end{aligned} \end{aligned}$$

$$\begin{aligned} \begin{aligned}&\texttt {(2.2)}~ \gamma _1=\gamma _{-1}=0,~\gamma _{-2}=-\frac{3 \alpha _1^2 \mu _2 \tau _2^2}{2 \mu _1 \tau _4} ,~\gamma _2= -\frac{6 \alpha _1^2 \mu _2 \tau _4}{\mu _1},~\gamma _0=\frac{4 \left( -\alpha _2 \eta \mu _3-4 \alpha _1^4 \mu _2 \tau _2-\alpha _1^2+\eta ^2\right) +\alpha _2^2 \mu _3^2}{8 \alpha _1^2 \mu _1}.\\ \end{aligned} \end{aligned}$$

$$\begin{aligned} \begin{aligned}&\texttt {(2.3)}~\gamma _1~=\gamma _{-1}~=\gamma _{-2}~=0,~\gamma _2= -\frac{6 \alpha _1^2 \mu _2 ~\tau _4}{\mu _1},~\text {and}~\gamma _0~=\frac{4 ~\left( -\alpha _2~ \eta \mu _3-4 \alpha _1^4~ \mu _2 \tau _2-\alpha _1^2+\eta ^2\right) +\alpha _2^2 \mu _3^2}{8 \alpha _1^2 \mu _1}.\\ \end{aligned} \end{aligned}$$

From the set (2.1), the following solutions are obtained under condition $\alpha _1^2\, \mu _1\ne 0$:

The singular soliton with $\tau _2<0$, and singular periodic solution with $\tau _2>0$ are described below:

12 $$\begin{aligned} P_{2.1,1}( \,x, y, t )= \frac{\frac{1}{4} \left( \alpha _2 \mu _3-2 \eta \right) {}^2-\alpha _1^2-2 \alpha _1^4 \mu _2 \tau _2 \left( 2-3 \coth ^2\left( \left( \alpha _1 x+\alpha _2 y-\eta t\right) \sqrt{-\frac{\tau _2}{2}}\right) \right) }{2 \alpha _1^2 \mu _1}, \end{aligned}$$

13 $$\begin{aligned} P_{2.1,2}( \,x, y, t )=\frac{\frac{1}{4} \left( \alpha _2 \mu _3-2 \eta \right) {}^2-\alpha _1^2-2 \alpha _1^4 \mu _2 \tau _2 \left( 3 \cot ^2\left( \left( \alpha _1 x+\alpha _2 y-\eta t\right) \sqrt{\frac{\tau _2}{2}} \right) +2\right) }{2 \alpha _1^2 \mu _1}. \end{aligned}$$

From the set (2.2), the following solutions are obtained under condition $\alpha _1^2\, \mu _1\ne 0$: The singular soliton with $\tau _2<0$, and singular periodic solution with $\tau _2>0$ are as follows:

14 $$\begin{aligned} P_{2.2,1}( \,x, y, t )= \frac{\frac{1}{4} \left( \alpha _2 \mu _3-2 \eta \right) {}^2-\alpha _1^2+8 \alpha _1^4 \mu _2 \tau _2 \left( 3 \text {csch}^2\left( \left( \alpha _1 x+\alpha _2 y-\eta t\right) \sqrt{-2 \tau _2}\right) +1\right) }{2 \alpha _1^2 \mu _1}, \end{aligned}$$

15 $$\begin{aligned} P_{2.2,2}( \,x, y, t )=\frac{\frac{1}{4} \left( \alpha _2 \mu _3-2 \eta \right) {}^2-\alpha _1^2+8 \alpha _1^4 \mu _2 \tau _2 \left( 1-3 \csc ^2\left( \left( \alpha _1 x+\alpha _2 y-\eta t\right) \sqrt{2 \tau _2}\right) \right) }{2 \alpha _1^2 \mu _1}. \end{aligned}$$

From the set (2.3), the following solutions are obtained under condition $\alpha _1^2\, \mu _1\ne 0$:

The dark soliton with $\tau _2<0$, and singular periodic solution with $\tau _2>0$ are as follows:

16 $$\begin{aligned} P_{2.3,1}( \,x, y, t )= \frac{\frac{1}{4} \left( \alpha _2 \mu _3-2 \eta \right) {}^2-\alpha _1^2+2 \alpha _1^4 \mu _2 \tau _2 \left( 3 \tanh ^2\left( \left( \alpha _1 x+\alpha _2 y-\eta t\right) \sqrt{-\frac{\tau _2}{2}}\right) -2\right) }{2 \alpha _1^2 \mu _1}. \end{aligned}$$

17 $$\begin{aligned} P_{2.3,2}( \,x, y, t )=\frac{\frac{1}{4} \left( \alpha _2 \mu _3-2 \eta \right) {}^2-\alpha _1^2-2 \alpha _1^4 \mu _2 \tau _2 \left( 3 \tan ^2\left( \left( \alpha _1 x+\alpha _2 y-\eta t\right) \sqrt{\frac{\tau _2}{2}} \right) +2\right) }{2 \alpha _1^2 \mu _1}. \end{aligned}$$

**Case** C: Upon $\tau _3=~\tau _4=~\tau _6=0$, the following outcomes are produced:

$$\begin{aligned} \begin{aligned} \texttt {(3.1)}~&\gamma _1=~\gamma _2=0,~\gamma _{-1}=-\frac{3 \alpha _1^2~ \mu _2 \tau _1}{\mu _1} ,~\gamma _0=\frac{4 \left( -\alpha _2 \eta \mu _3+\alpha _1^4 \left( -\mu _2\right) \tau _2-\alpha _1^2+\eta ^2\right) +\alpha _2^2 \mu _3^2}{8 \alpha _1^2 \mu _1},~ \tau _0=\frac{\tau _1^2}{4 \tau _2} ,\\&\gamma _{-2}=-\frac{3 \alpha _1^2 \mu _2 \tau _1^2}{2 \mu _1 \tau _2}.\\ \end{aligned} \end{aligned}$$

$$\begin{aligned} \begin{aligned}&\texttt {(3.2)}~\gamma _1=\gamma _{-1}=\gamma _2=0,~\gamma _{-2}=-\frac{6 \alpha _1^2 \mu _2 \tau _0}{\mu _1},~\gamma _0=\frac{4 \left( -\alpha _2 \eta \mu _3-4 \alpha _1^4 \mu _2 \tau _2-\alpha _1^2+\eta ^2\right) +\alpha _2^2 \mu _3^2}{8 \alpha _1^2 \mu _1},~\tau _1=0.\\ \end{aligned} \end{aligned}$$

From the set (3.1), the exponential solution is obtained under condition $\alpha _1^2\, \mu _1\ne 0$ with $\tau _2>0$, $\tau _0=\frac{\tau _1^2}{4 \tau _2}$:

18 $$\begin{aligned} P_{3.1,1}( \,x, y, t )=\frac{\frac{1}{4} \left( \alpha _2 \mu _3-2 \eta \right) {}^2-\alpha _1^2-\alpha _1^4 \mu _2 \tau _2 \left( \frac{24 \tau _1 \tau _2 \exp ^{\left( \alpha _1 x+\alpha _2 y-\eta t\right) \sqrt{\tau _2} }}{\left( \tau _1-2 \tau _2 \exp ^{ \left( \alpha _1 x+\alpha _2 y-\eta t\right) \sqrt{\tau _2} }\right) {}^2}+1\right) }{2 \alpha _1^2 \mu _1}, \end{aligned}$$

From the set (3.2), the following are obtained under condition $\alpha _1^2\, \mu _1\ne 0$:

The singular soliton with $\tau _2>0$,  $\tau _1=0$, the singular periodic solution with $\tau _2<0$,  $\tau _1=0$, and exponential solution with $\tau _2<0$,  $\tau _0=\frac{\tau _1^2}{4 \tau _2}$ are as follows .

19 $$\begin{aligned} P_{3.2,1}( \,x, y, t )=\frac{\frac{1}{4} \left( \alpha _2 \mu _3-2 \eta \right) {}^2-\alpha _1^2-4 \alpha _1^4 \mu _2 \tau _2 \left( 3 \text {csch}^2\left( \left( \alpha _1 x+\alpha _2 y-\eta t\right) \sqrt{\tau _2} \right) +1\right) }{2 \alpha _1^2 \mu _1}, \end{aligned}$$

20 $$\begin{aligned} P_{3.2,2}( \,x, y, t )=\frac{\frac{1}{4} \left( \alpha _2 \mu _3-2 \eta \right) {}^2-\alpha _1^2+4 \alpha _1^4 \mu _2 \tau _2 \left( 3 \csc ^2\left( \left( \alpha _1 x+\alpha _2 y-\eta t\right) \sqrt{-\tau _2} \right) -1\right) }{2 \alpha _1^2 \mu _1}, \end{aligned}$$

21 $$\begin{aligned} P_{3.2,3}( \,x, y, t )=\frac{\frac{1}{4} \left( \alpha _2 \mu _3-2 \eta \right) {}^2-\alpha _1^2-4 \alpha _1^4 \mu _2 \left( 3 \tau _0 \exp ^{-2 \left( \alpha _1 x+\alpha _2 y-\eta t\right) \sqrt{\tau _2} }+\tau _2\right) }{2 \alpha _1^2 \mu _1}. \end{aligned}$$

**Case** D: Upon $\tau _0=~\tau _1=~\tau _6=0$, the following outcomes are produced:

$$\begin{aligned} \begin{aligned}&\texttt {(4.1)}~\gamma _1=~\gamma _{-1}=~\gamma _{-2}=0,~\gamma _2=-\frac{6 \alpha _1^2 \mu _2~ \tau _4}{\mu _1},~\gamma _0=\frac{4 \left( -\alpha _2 ~\eta \mu _3-4 \alpha _1^4~ \mu _2 ~\tau _2-\alpha _1^2+~\eta ^2\right) +~\alpha _2^2 \mu _3^2}{8 \alpha _1^2 \mu _1},~\tau _3=0.\\ \end{aligned} \end{aligned}$$

$$\begin{aligned} \begin{aligned} \texttt {(4.2)}~&\gamma _{-1}=~\gamma _{-2}=0,~\gamma _1=\mp \frac{6 \alpha _1^2 \mu _2 \sqrt{\tau _2 \tau _4}}{\mu _1},~\gamma _2=\mp \frac{6 \alpha _1^2 \mu _2 \tau _4}{\mu _1},~\gamma _0=\frac{4 \left( -\alpha _2 \eta \mu _3-\mu _2\alpha _1^4 \tau _2-\alpha _1^2+\eta ^2\right) +\alpha _2^2 \mu _3^2}{8 \alpha _1^2 \mu _1},~\\&\tau _3=\mp 2\sqrt{\tau _2 \tau _4}.\\ \end{aligned} \end{aligned}$$

From the set (4.1), the following are obtained under conditions $\alpha _1^2\, \mu _1\ne 0$, and $\tau _3^2\ne 4 \tau _2 \,\tau _4$:

The singular soliton with $\tau _2>0$, and the singular periodic solution with $\tau _2<0$ are described below:

22 $$\begin{aligned} P_{4.1,1}( \,x, y, t )=\frac{\frac{1}{4} \left( \alpha _2 \mu _3-2 \eta \right) {}^2-\alpha _1^2-4 \alpha _1^4 \mu _2 \tau _2 \left( 3 \text {csch}^2\left( 1+\left( \alpha _1 x+\alpha _2 y-\eta t\right) \sqrt{\tau _2} \right) \right) }{2 \alpha _1^2 \mu _1}, \end{aligned}$$

23 $$\begin{aligned} P_{4.1,2}( \,x, y, t )=\frac{\frac{1}{4} \left( \alpha _2 \mu _3-2 \eta \right) {}^2-\alpha _1^2-4 \alpha _1^4 \mu _2 \tau _2 \left( 1-3 \csc ^2\left( \left( \alpha _1 x+\alpha _2 y-\eta t\right) \sqrt{-\tau _2}\right) \right) }{2 \alpha _1^2 \mu _1}. \end{aligned}$$

From the set (4.2), the following are obtained under conditions $\alpha _1^2\, \mu _1\ne 0$, and $\tau _3^2\,=\, 4 \tau _2\, \tau _4$:

The dark soliton with $\tau _2>0$, and the singular soliton with $\tau _2>0$ are as follows:

24 $$\begin{aligned} P_{4.2,1}( \,x, y, t )=\mp \frac{\frac{1}{4} \left( \alpha _2 \mu _3-2 \eta \right) {}^2-\alpha _1^2+\alpha _1^4 \mu _2 \tau _2 \left( 2-3 \tanh ^2\left( \frac{1}{2} \left( \alpha _1 x+\alpha _2 y-\eta t\right) \sqrt{\tau _2}\right) \right) }{2 \alpha _1^2 \mu _1}, \end{aligned}$$

25 $$\begin{aligned} P_{4.2,2}( \,x, y, t )=\frac{\frac{1}{4} \left( \alpha _2 \mu _3-2 \eta \right) {}^2-\alpha _1^2+\alpha _1^4 \mu _2 \tau _2 \left( 2-3 \coth ^2\left( \frac{1}{2} \left( \alpha _1 x+\alpha _2 y-\eta t\right) \sqrt{\tau _2}\right) \right) }{2 \alpha _1^2 \mu _1}. \end{aligned}$$

**Case** E: Upon $\tau _1=~\tau _3=~\tau _6=0$, the following outcomes are produced:

$$\begin{aligned} \begin{aligned}&\texttt {(5.1)}~\gamma _1=~\gamma _{-1}~=\gamma _2~=0,~\gamma _{-2}=-\frac{6 \alpha _1^2 \mu _2 \tau _0}{\mu _1},~\gamma _0=\frac{4 \left( -\alpha _2 \eta \mu _3-4 \alpha _1^4 \mu _2 \tau _2-\alpha _1^2+\eta ^2\right) +\alpha _2^2 \mu _3^2}{8 \alpha _1^2 \mu _1}.\\ \end{aligned} \end{aligned}$$

$$\begin{aligned} \begin{aligned}&\texttt {(5.2)}~\gamma _1=~\gamma _{-1}~=\gamma _{-2}~=0,~\gamma _{2}=-\frac{6 \alpha _1^2 \mu _2 \tau _4}{\mu _1},~\gamma _0=\frac{4 \left( -\alpha _2 \eta \mu _3-4 \alpha _1^4 \mu _2 \tau _2-\alpha _1^2+\eta ^2\right) +\alpha _2^2 \mu _3^2}{8 \alpha _1^2 \mu _1}.\\ \end{aligned} \end{aligned}$$

$$\begin{aligned} \begin{aligned} \texttt {(5.3)}~&\gamma _1=~\gamma _{-1}~==0,~\gamma _{2}=-\frac{6 \alpha _1^2 \mu _2 \tau _4}{\mu _1},~ \gamma _0=\frac{4~ \left( -\alpha _2~ \eta ~ \mu _3-4 ~\alpha _1^4 \mu _2 ~\tau _2-\alpha _1^2+~\eta ^2\right) +\alpha _2^2 ~\mu _3^2}{8 \alpha _1^2 \mu _1},\\&\gamma _{-2}=-\frac{6 \alpha _1^2 \mu _2 \tau _0}{\mu _1},~.\\ \end{aligned} \end{aligned}$$

According to the set of responses (5.1), Eq. (1) provide exact solutions that may be represented as:

**Case** (i): If $\tau _0=1,~\tau _2=-1-n^2,~\tau _4=n^2,~ \alpha _1^2 \mu _1\ne 0,~\text {and} ~0\le n\le 1$, the Jacobi elliptic functional *JEF* solutions are as follows:

26 $$\begin{aligned} P_{5.1}( \,x, y, t )=\frac{\frac{1}{4} \left( \alpha _2 \mu _3-2 \eta \right) {}^2-\alpha _1^2+4 \alpha _1^4 \mu _2 \left( 1+n^2-\frac{3}{\text {sn}{}^2\left( \alpha _1 x+ \alpha _2 y-\eta t\right) }\right) }{2 \alpha _1^2 \mu _1}, \end{aligned}$$

or,

27 $$\begin{aligned} P_{5.2}( \,x, y, t )=\frac{\frac{1}{4} \left( \alpha _2 \mu _3-2 \eta \right) {}^2-\alpha _1^2-4 \alpha _1^4 \mu _2 \left( \frac{3 \tau _0}{\text {cd}{}^2\left( \alpha _1 x+ \alpha _2 y-\eta t\right) }+\tau _2\right) }{2 \alpha _1^2 \mu _1}. \end{aligned}$$

Setting $n = 0$ in Eqs. (26) and (27) results in singular periodic solutions as follows:

28 $$\begin{aligned} P_{5.1,1}( \,x, y, t )=\frac{\frac{1}{4} \left( \alpha _2 \mu _3-2 \eta \right) {}^2-\alpha _1^2+4 \alpha _1^4 \mu _2 \left( 1-3 \csc ^2\left( \alpha _1 x+\alpha _2 y -\eta t\right) \right) }{2 \alpha _1^2 \mu _1}, \end{aligned}$$

29 $$\begin{aligned} P_{5.2,1}( \,x, y, t )=\frac{\frac{1}{4} \left( \alpha _2 \mu _3-2 \eta \right) {}^2-\alpha _1^2+4 \alpha _1^4 \mu _2 \left( 1-3 \sec ^2\left( \alpha _1 x+\alpha _2 y -\eta t\right) \right) }{8 \alpha _1^2 \mu _1}. \end{aligned}$$

Setting $n = 1$ in Eq. (26) yields a singular soliton solution as shown below:

30 $$\begin{aligned} P_{5.1,2}( \,x, y, t )=\frac{\frac{1}{4} \left( \alpha _2 \mu _3-2 \eta \right) {}^2-\alpha _1^2+4 \alpha _1^4 \mu _2 \left( 2-3 \coth ^2\left( \alpha _1 x+\alpha _2 y -\eta t\right) \right) }{2 \alpha _1^2 \mu _1}. \end{aligned}$$

**Case** (ii): If $\tau _0=n^2-1,~\tau _2=2-n^2,~\tau _4=-1,~ \alpha _1^2 \mu _1\ne 0~\text {and}~ ~0\le n\le 1$, the *JEF* solution is described below:

31 $$\begin{aligned} P_{5.3}( \,x, y, t )=\frac{\frac{1}{4} \left( \alpha _2 \mu _3-2 \eta \right) {}^2-\alpha _1^2-4 \alpha _1^4 \mu _2 \left( \frac{3 \tau _0}{\text {cd}{}^2\left( \alpha _1 x + \alpha _2 y-\eta t \right) }+\tau _2\right) }{2 \alpha _1^2 \mu _1}. \end{aligned}$$

**Case** (iii): If $\tau _0=-n^2,~\tau _2=2n^2-1,~\tau _4=1-n^2,~ \alpha _1^2 \mu _1\ne 0,~\text {and} ~0\le n\le 1$, the *JEF* solution is described below:

32 $$\begin{aligned} P_{5.4}( \,x, y, t )=\frac{\frac{1}{4} \left( \alpha _2 \mu _3-2 \eta \right) {}^2-\alpha _1^2+4 \alpha _1^4 \mu _2 \left( 3 n^2 \text {cn}{}^2\left( \alpha _1 x+ \alpha _2 y-\eta t \right) -2 n^2+1\right) }{2 \alpha _1^2 \mu _1}. \end{aligned}$$

Setting $n = 1$ in Eq. (32) degenerates a bright soliton as follows:

33 $$\begin{aligned} P_{5.4,1}( \,x, y, t )=\frac{\frac{1}{4} \left( \alpha _2 \mu _3-2 \eta \right) {}^2-\alpha _1^2-4 \alpha _1^4 \mu _2 \left( 1-3 \text {sech}^2\left( \alpha _1 x+\alpha _2 y -\eta t\right) \right) }{2 \alpha _1^2 \mu _1}. \end{aligned}$$

**Case** (iv): If $\tau _0=-1,~\tau _2=2-n^2,~\tau _4=n^2-1,~ \alpha _1^2 \mu _1\ne 0,~\text {and} ~0\le n\le 1$, the *JEF* solution is detailed below:

34 $$\begin{aligned} P_{5.5}( \,x, y, t )=\frac{\frac{1}{4} \left( \alpha _2 \mu _3-2 \eta \right) {}^2-\alpha _1^2+4 \alpha _1^4 \mu _2 \left( 3 \text {dn}{}^2\left( \alpha _1 x + \alpha _2 y-\eta t \right) +n^2-2\right) }{2 \alpha _1^2 \mu _1}. \end{aligned}$$

Setting $n = 1$ in Eq. (34) results a bright soliton:

35 $$\begin{aligned} P_{5.5,1}( \,x, y, t )=\frac{\frac{1}{4} \left( \alpha _2 \mu _3-2 \eta \right) {}^2-\alpha _1^2-4 \alpha _1^4 \mu _2 \left( 1-3 \text {sech}^2\left( \alpha _1 x+\alpha _2 y -\eta t\right) \right) }{2 \alpha _1^2 \mu _1}. \end{aligned}$$

**Case** (v): If $\tau _0=1,~\tau _2=2-4n^2,~\tau _4=1,~ \alpha _1^2 \mu _1\ne 0,~\text {and} ~0\le n\le 1$, the *JEF* solution is described below:

36 $$\begin{aligned} P_{5.6}( \,x, y, t )=\frac{\frac{1}{4} \left( \alpha _2 \mu _3-2 \eta \right) {}^2-\alpha _1^2-4 \alpha _1^4 \mu _2 \left( \frac{3 \text {cn}{}^2\left( \alpha _1 x +\alpha _2 y -\eta t \right) }{\text {dn}{}^2 \left( \alpha _1 x + \alpha _2 y -\eta t \right) \text {sn}{}^2\left( \alpha _1 x+ \alpha _2 y-\eta t \right) }+\tau _2\right) }{2 \alpha _1^2 \mu _1}. \end{aligned}$$

Setting $n = 0$ and $n = 1$ in Eq. (36) produces singular periodic and singular soliton:

37 $$\begin{aligned} P_{5.6,1}( \,x, y, t )=\frac{\frac{1}{4} \left( \alpha _2 \mu _3-2 \eta \right) {}^2-\alpha _1^2-4 \alpha _1^4 \mu _2 \left( 3 \cot ^2\left( \alpha _1 x+\alpha _2 y -\eta t\right) +2\right) }{2 \alpha _1^2 \mu _1}, \end{aligned}$$

38 $$\begin{aligned} P_{5.6,2}( \,x, y, t )=\frac{\frac{1}{4} \left( \alpha _2 \mu _3-2 \eta \right) {}^2-\alpha _1^2+4 \alpha _1^4 \mu _2 \left( 2-3 \coth ^2\left( \alpha _1 x+\alpha _2 y -\eta t\right) \right) }{2 \alpha _1^2 \mu _1}. \end{aligned}$$

**Case** (vi): If $\tau _0=n^4-2 n^3+n^2,~\tau _2=-\frac{4}{n},~\tau _4=-n^2+6 n-1,~ \alpha _1^2 \mu _1\ne 0,~\text {and} ~0< n\le 1$, the *JEF* solution is described below:

39 $$\begin{aligned} P_{5.7}( \,x, y, t )=\frac{\frac{1}{4} \left( \alpha _2 \mu _3-2 \eta \right) {}^2-\alpha _1^2-\frac{12 \alpha _1^4 \mu _2 (n-1)^2 \left( \text {dn}{}^2\left( \alpha _1 x +\alpha _2 y -\eta t \right) -2\right) {}^2}{\text {cn}{}^2\left( x \alpha _1+y \alpha _2-\eta t \right) {}^2 \text {dn}{}^2\left( \alpha _1 x + \alpha _2 y -\eta t \right) }+\frac{16 \alpha _1^4 \mu _2}{n}}{2 \alpha _1^2 \mu _1}. \end{aligned}$$

**Case** (vii): If $\tau _0=\frac{1}{4},~\tau _2=\frac{1}{2} \left( n^2-2\right) ,~\tau _4=\frac{n^4}{4},~ \alpha _1^2\,\mu _1\ne 0,~\text {and} ~0\le n\le 1$, the *JEF* solutions are as follows:

40 $$\begin{aligned} \begin{aligned}&P_{5.8}( \,x, y, t )=\frac{\frac{12 \alpha _1^4 \mu _2 \left( n^2 -1-\text {dn}\left( -\alpha _1 x -\alpha _2 y +\eta t \right) \left( \text {dn}\left( -\alpha _1 x -\alpha _2 y +\eta t \right) +2 \sqrt{1-n^2}\right) \right) +\left( \alpha _2 \mu _3-2 \eta \right) {}^2-4 \alpha _1^2-8 \alpha _1^4 \mu _2 \left( n^2-2\right) }{\text {cn}{}^2\left( -\alpha _1 x -\alpha _2 y +\eta t \right) }}{8 \alpha _1^2 \mu _1},\\ \end{aligned} \end{aligned}$$

or,

41 $$\begin{aligned} P_{5.9}( \,x, y, t )=\frac{\frac{1}{4} \left( \alpha _2 \mu _3-2 \eta \right) {}^2-\alpha _1^2-\frac{3 \alpha _1^4 \mu _2 \left( \text {dn}\left( \alpha _1 x +\alpha _2 y -\eta t \right) +1\right) {}^2}{\text {sn}{}^2\left( \alpha _1 x +\alpha _2 y -\eta t \right) }-2 \alpha _1^4 \mu _2 \left( n^2-2\right) }{2 \alpha _1^2 \mu _1}. \end{aligned}$$

Setting $n = 0$ in Eqs. (40) and (41) yield singular periodic solutions as follows::

42 $$\begin{aligned} P_{5.8,1}( \,x, y, t ) =\frac{\frac{1}{4} \left( \alpha _2 \mu _3-2 \eta \right) {}^2-\alpha _1^2+4 \alpha _1^4 \mu _2 \left( 1-3 \sec ^2\left( -\alpha _1 x-\alpha _2 y+ \eta t\right) \right) }{2 \alpha _1^2 \mu _1}, \end{aligned}$$

43 $$\begin{aligned} P_{5.9,1}( \,x, y, t ) =\frac{\frac{1}{4} \left( \alpha _2 \mu _3-2 \eta \right) {}^2-\alpha _1^2+4 \alpha _1^4 \mu _2 \left( 1-3 \csc ^2\left( -\alpha _1 x-\alpha _2 y+\eta t\right) \right) }{2 \alpha _1^2 \mu _1}. \end{aligned}$$

Setting $n = 1$ in Eq. (41) produces a hyperbolic wave solution as follows::

44 $$\begin{aligned} P_{5.9,2}( \,x, y, t )=\frac{\frac{\left( \alpha _2 \mu _3-2 \eta \right) {}^2}{2}-2 \alpha _1^2-\mu _2\alpha _1^4 \left( \cosh \left( -\alpha _1 x-\alpha _2 y+\eta t\right) +5\right) \text {csch}^2\left( \frac{1}{2} \left( \alpha _1 x+\alpha _2 y-\eta t\right) \right) }{4 \alpha _1^2 \mu _1}. \end{aligned}$$

According to the set of replies (5.2), Eq. (1) yield exact solutions that may be expressed as:

**Case** (i): If $\tau _0=1,~\tau _2=-1-n^2,~\tau _4=n^2,~ \alpha _1^2 \mu _1\ne 0,~\text {and} ~0\le n\le 1$, the *JEF* solutions are described below:

45 $$\begin{aligned} P_{5.10}( \,x, y, t )=\frac{\left( \alpha _2 \mu _3-2 \eta \right) {}^2-4 \alpha _1^2+16 \alpha _1^4 \mu _2 \left( 1+n^2-3 n^2 \text {sn}{}^2\left( \alpha _1 x +\alpha _2 y - \eta t\right) \right) }{8 \alpha _1^2 \mu _1}, \end{aligned}$$

or,

46 $$\begin{aligned} P_{5.11}( \,x, y, t )=\frac{\frac{1}{4} \left( \alpha _2 \mu _3-2 \eta \right) {}^2-\alpha _1^2+4\alpha _1^4\mu _2\left( 1+n^2-3n^2 \text {cd}{}^2\left( \alpha _1 x +\alpha _2 y - \eta t\right) \right) }{2 \alpha _1^2 \mu _1}. \end{aligned}$$

Setting $n = 1$ in Eq. (45) produces a dark soliton as follows::

47 $$\begin{aligned} P_{5.10,1}( \,x, y, t )=\frac{\frac{1}{4} \left( \alpha _2 \mu _3-2 \eta \right) {}^2-\alpha _1^2+4 \alpha _1^4 \mu _2 \left( 2-3 \tanh ^2\left( \alpha _1 x+\alpha _2 y - \eta t\right) \right) }{2 \alpha _1^2 \mu _1}. \end{aligned}$$

**Case** (ii): If $\tau _0=n^2-1,~\tau _2=2-n^2,~\tau _4=-1,~ \alpha _1^2 \mu _1\ne 0~\text {and} ~0\le \, n\le 1$, the *JEF* solution is described below:

48 $$\begin{aligned} P_{5.12}( \,x, y, t )=\frac{\frac{1}{4} \left( \alpha _2 \mu _3-2 \eta \right) {}^2-\alpha _1^2+4 \alpha _1^4 \mu _2 \left( 3 \text {dn}{}^2\left( -\alpha _1 x -\alpha _2y +\eta t\right) +n^2-2\right) }{2 \alpha _1^2 \mu _1}. \end{aligned}$$

Setting $n = 1$ in Eq. (48) degenerates a bright soliton as follows::

49 $$\begin{aligned} P_{5.12,1}( \,x, y, t )=\frac{\frac{1}{4} \left( \alpha _2 \mu _3-2 \eta \right) {}^2-\alpha _1^2-4 \alpha _1^4 \mu _2 \left( 1-3 \text {sech}^2\left( \alpha _1 x+\alpha _2 y- \eta t\right) \right) }{2 \alpha _1^2 \mu _1}. \end{aligned}$$

**Case** (iii): If $\tau _0=-n^2,~\tau _2=2n^2-1,~\tau _4=1-n^2,~ \alpha _1^2 \mu _1\ne 0,~\text {and} ~0\le n\le 1$, the *JEF* solution is described below:

50 $$\begin{aligned} P_{5.13}( \,x, y, t )=\frac{\frac{1}{4} \left( \alpha _2 \mu _3-2 \eta \right) {}^2-\alpha _1^2+4 \alpha _1^4 \mu _2 \left( 3 \left( n^2-1\right) \text {nc}{}^2\left( \alpha _1 x +\alpha _2 y -\eta t \right) -2 n^2+1\right) }{2 \alpha _1^2 \mu _1}. \end{aligned}$$

Setting $n = 0$ in Eq. (50) gives a singular periodic wave solution as follows::

51 $$\begin{aligned} P_{5.13,1}( \,x, y, t )=\frac{\frac{1}{4} \left( \alpha _2 \mu _3-2 \eta \right) {}^2-\alpha _1^2+4 \alpha _1^4 \mu _2 \left( 1-3 \sec ^2\left( -\alpha _1 x-\alpha _2 y+ \eta t\right) \right) }{2 \alpha _1^2 \mu _1}. \end{aligned}$$

**Case** (iv): If $\tau _0=-1,~\tau _2=2-n^2,~\tau _4=n^2-1,~ \alpha _1^2 \mu _1\ne 0,~\text {and} ~0\le n\le 1$, the *JEF* solution is described below:

52 $$\begin{aligned} P_{5.14}( \,x, y, t )=\frac{\frac{1}{4} \left( \alpha _2 \mu _3-2 \eta \right) {}^2-\alpha _1^2+4 \alpha _1^4 \mu _2 \left( \frac{3-3 n^2}{\text {dn}{}^2\left( - \alpha _1 x- \alpha _2 y + \eta t\right) }+n^2-2\right) }{2 \alpha _1^2 \mu _1}. \end{aligned}$$

**Case** (v): If $\tau _0=1,~\tau _2=2-4n^2,~\tau _4=1,~ \alpha _1^2 \mu _1\ne 0,~\text {and} ~0\le n\le 1$, the *JEF* solution is as follows:

53 $$\begin{aligned} \begin{aligned}&P_{5.15}( \,x, y, t )=\frac{2 \alpha _1^4 \mu _2 \left( 3 \text {dn}{}^2\left( -\alpha _1 x-\alpha _2 y+\eta t\right) {}^2 \text {nc}{}^2\left( - \alpha _1 x-\alpha _2 y+\eta t\right) \text {sn}{}^2\left( -\alpha _1 x -\alpha _2 y +\eta t\right) +\tau _2\right) }{- \alpha _1^2 \mu _1}\\&-\frac{\alpha _1^2}{2 \alpha _1^2 \mu _1}+\frac{\left( \alpha _2 \mu _3-2 \eta \right) {}^2}{8 \alpha _1^2 \mu _1}.\\ \end{aligned} \end{aligned}$$

Setting $n = 0$ and $n = 1$ in Eq. (53) produces singular periodic and dark soliton solutions as follows:

54 $$\begin{aligned} P_{5.15,1}( \,x, y, t )=\frac{\frac{1}{4} \left( \alpha _2 \mu _3-2 \eta \right) {}^2-\alpha _1^2-4 \alpha _1^4 \mu _2 \left( 2+3 \tan ^2\left( -\alpha _1 x-\alpha _2 y+\eta t\right) \right) }{2 \alpha _1^2 \mu _1}, \end{aligned}$$

55 $$\begin{aligned} P_{5.15,2}( \,x, y, t )=\frac{\frac{1}{4} \left( \alpha _2 \mu _3-2 \eta \right) {}^2-\alpha _1^2+4 \alpha _1^4 \mu _2 \left( 2-3 \tanh ^2\left( \alpha _1 x+\alpha _2 y- \eta t\right) \right) }{2 \alpha _1^2 \mu _1}. \end{aligned}$$

**Case** (vi): If $\tau _0=n^4-2 n^3+n^2,~\tau _2=-\frac{4}{n},~\tau _4=-n^2+6 n-1,~ \alpha _1^2 \mu _1\ne 0,~\text {and} ~0< n\le 1$, the *JEF* solution is described below:

56 $$\begin{aligned} P_{5.16}( \,x, y, t )=\frac{\frac{1}{4} \left( \alpha _2 \mu _3-2 \eta \right) {}^2-\alpha _1^2+\frac{16 \alpha _1^4 \mu _2 \left( \frac{3 ((n-6) n+1) n^3 \text {cn}{}^2\left( -\alpha _1 x-\alpha _2 y+\eta t\right) \text {dn}{}^2\left( -\alpha _1 x-\alpha _2 y+\eta t\right) }{4 \left( \text {dn}{}^2\left( -\alpha _1 x-\alpha _2 y+\eta t\right) -2\right) {}^2}+1\right) }{n}}{2 \alpha _1^2 \mu _1}. \end{aligned}$$

Setting $n = 1$ in Eq. (56) gives a hyperbolic wave solution:

57 $$\begin{aligned} P_{5.16,1}( \,x, y, t )=\frac{\frac{\left( \alpha _2 \mu _3-2 \eta \right) {}^2}{4} -\alpha _1^2+8 \alpha _1^4 \mu _2 \left( \cosh \left( -4 \alpha _1 x-4 \alpha _2 y+4\eta t\right) -5\right) \text {sech}^2\left( -2 \alpha _1 x-2 \alpha _2 y+2 \eta t\right) }{2 \alpha _1^2 \mu _1}. \end{aligned}$$

**Case** (vii): If $\tau _0=\frac{1}{4},~\tau _2=\frac{1}{2} \left( n^2-2\right) ,~\tau _4=\frac{n^4}{4},~ \alpha _1^2\,\mu _1\ne 0,~\text {and} ~0\le n\le 1$, the *JEF* solutions are described below:

58 $$\begin{aligned} P_{5.17}( \,x, y, t )=\frac{\frac{1}{4} \left( \alpha _2 \mu _3-2 \eta \right) {}^2-\alpha _1^2-\mu _2\alpha _1^4 \left( \frac{3 n^4 \text {cn}{}^2\left( -\alpha _1 x- \alpha _2 y+\eta t\right) }{\left( \text {dn}\left( -\alpha _1 x- \alpha _2 y+\eta t\right) +\sqrt{1-n^2}\right) {}^2}+4 \tau _2\right) }{2 \alpha _1^2 \mu _1}, \end{aligned}$$

or,

59 $$\begin{aligned} P_{5.18}( \,x, y, t )=\frac{\frac{1}{4} \left( \alpha _2 \mu _3-2 \eta \right) {}^2-\alpha _1^2+\alpha _1^4 \mu _2 \left( -\left( \frac{3 n^4 \text {sn}{}^2\left( -\alpha _1 x- \alpha _2 y+\eta t\right) }{\left( 1+\text {dn}\left( \alpha _1 x-\alpha _2 y+\eta t\right) \right) {}^2}+4 \tau _2\right) \right) }{2 \alpha _1^2 \mu _1}. \end{aligned}$$

Setting $n = 1$ in Eq. (59) gives a dark soliton as follows:

60 $$\begin{aligned} P_{5.18,1}( \,x, y, t )=\frac{\frac{1}{4} \left( \alpha _2 \mu _3-2 \eta \right) {}^2-\alpha _1^2+4 \alpha _1^4 \mu _2 \left( 2-3 \tanh ^2\left( \alpha _1 x+\alpha _2 y - \eta t\right) \right) }{2 \alpha _1^2 \mu _1}. \end{aligned}$$

According to the set of solution (5.3), Eq. (1) degenerates exact solutions, which may be represented as:

**Case** (i): If $\tau _0=1,~\tau _2=-1-n^2,~\tau _4=n^2,~ \alpha _1^2 \mu _1\ne 0,~\text {and} ~0\le n\le 1$, the *JEF* solutions are as follows:

61 $$\begin{aligned} P_{5.19}( \,x, y, t )=\frac{\frac{\left( \alpha _2 \mu _3-2 \eta \right) {}^2}{4} -\alpha _1^2-12 \alpha _1^4 \mu _2 \left( n^2 \text {sn}{}^2\left( - \alpha _1 x-\alpha _2 y+\eta t\right) +\frac{1}{\text {sn}{}^2\left( - \alpha _1-\alpha _2 y+\eta t\right) }+\frac{\tau _2}{3}\right) }{2 \alpha _1^2 \mu _1}, \end{aligned}$$

or,

62 $$\begin{aligned} P_{5.20}( \,x, y, t )=\frac{\frac{\left( \alpha _2 \mu _3-2 \eta \right) {}^2}{4} -\alpha _1^2-4 \alpha _1^4 \mu _2 \left( 3 n^2 \text {cd}{}^2\left( \alpha _1 x + \alpha _2 y-\eta t \right) +\frac{3}{\text {cd}{}^2\left( \alpha _1 x+ \alpha _2 y-\eta t\right) }+\tau _2\right) }{2 \alpha _1^2 \mu _1}. \end{aligned}$$

Setting $n = 1$ in Eq. (61) yields a combo singular-dark soliton as follows:

63 $$\begin{aligned} \begin{aligned}&P_{5.19,1}( \,x, y, t )=\frac{\frac{\left( \alpha _2 \mu _3-2 \eta \right) {}^2}{4}- \alpha _1^2+\frac{4 \alpha _1^4 \mu _2}{3} \left( \frac{2}{3}-\tanh ^2\left( -\alpha _1 x-\alpha _2 y+\eta t\right) -\coth ^2\left( -\alpha _1 x-\alpha _2 y+\eta t\right) \right) }{2 \alpha _1^2 \mu _1}.\\ \end{aligned} \end{aligned}$$

Setting $n = 0$ in Eq. (62) produces a singular periodic wave solutions as follows:

64 $$\begin{aligned} P_{5.20,1}( \,x, y, t )=\frac{\frac{1}{4} \left( \alpha _2 \mu _3-2 \eta \right) {}^2-\alpha _1^2+4 \alpha _1^4 \mu _2 \left( 1-3 \sec ^2\left( -\alpha _1 x-\alpha _2 y+\eta t\right) \right) }{2 \alpha _1^2 \mu _1}. \end{aligned}$$

**Case** (ii): If $\tau _0=n^2-1,~\tau _2=2-n^2,~\tau _4=-1,~ \alpha _1^2 \mu _1\ne 0,~\text {and} ~0\le n\le 1$, the *JEF* solution is described below:

65 $$\begin{aligned} P_{5.21}( \,x, y, t )=\frac{\frac{\left( \alpha _2 \mu _3-2 \eta \right) {}^2}{4} -\alpha _1^2-4 \alpha _1^4 \mu _2 \left( -\frac{3 \tau _0}{\text {dn}{}^2\left( -\alpha _1 x-\alpha _2 y+\eta t\right) {}^2}+3 \text {dn}{}^2\left( - \alpha _1 x- \alpha _2 y+\eta t\right) +\tau _2\right) }{2 \alpha _1^2 \mu _1}. \end{aligned}$$

**Case** (iii): If $\tau _0=-n^2,~\tau _2=2n^2-1,~\tau _4=1-n^2,~ \alpha _1^2 \mu _1\ne 0~\text {and} ~0\le n\le 1$, the *JEF* solution is described below:

66 $$\begin{aligned} \begin{aligned}&P_{5.22}( \,x, y, t )=\frac{-\alpha _1^2+4 \alpha _1^4 \mu _2 \left( 3n^2 \text {cn}^2\left( -\alpha _1 x-\alpha _2 y+\eta t\right) +\frac{3 \left( n^2-1\right) }{\text {cn}^2\left( -\alpha _1 x-\alpha _2 y+\eta t\right) }-2 n^2+1\right) }{2 \alpha _1^2 \mu _1}\\&\frac{\left( \alpha _2 \mu _3-2 \eta \right) {}^2}{8 \alpha _1^2 \mu _1}.\\ \end{aligned} \end{aligned}$$

We are setting $n = 0$ and $n = 1$ in Eq. (66) gives singular periodic and bright soliton solutions as follows:

67 $$\begin{aligned} P_{5.22,1}( \,x, y, t )=\frac{\frac{1}{4} \left( \alpha _2 \mu _3-2 \eta \right) {}^2-\alpha _1^2+4 \alpha _1^4 \mu _2 \left( 1-3 \sec ^2\left( -\alpha _1 x-\alpha _2 y+ \eta t\right) \right) }{2 \alpha _1^2 \mu _1}, \end{aligned}$$

68 $$\begin{aligned} P_{5.22,2}( \,x, y, t )=\frac{\frac{1}{4} \left( \alpha _2 \mu _3-2 \eta \right) {}^2-\alpha _1^2+4 \alpha _1^4 \mu _2 \left( 3 \text {sech}{}^2\left( -\alpha _1 x-\alpha _2 y+\eta t\right) -1\right) }{2 \alpha _1^2 \mu _1}. \end{aligned}$$

**Case** (iv): If $\tau _0=-1,~\tau _2=2-n^2,~\tau _4=n^2-1,~ \alpha _1^2 \mu _1\ne 0,~\text {and} ~0\le n\le 1$, the *JEF* solution is described below:

69 $$\begin{aligned} P_{5.23}( \,x, y, t )=\frac{\frac{1}{4} \left( \alpha _2 \mu _3-2 \eta \right) {}^2-\alpha _1^2+\frac{4 \alpha _1^4 \mu _2 \left( 3 \text {dn}^4\left( -\alpha _1 x-\alpha _2 y+\eta t\right) +\text {dn}^2\left( -\alpha _1 x-\alpha _2 y+\eta t\right) \left( n^2-2\right) -3 n^2+3\right) }{\text {dn}\left( \left. -\alpha _1 x -\alpha _2 y +\eta t \right| n\right) {}^2}}{2 \alpha _1^2 \mu _1}. \end{aligned}$$

A bright soliton solution is obtained by putting $n = 1$ in Eq. (69).

70 $$\begin{aligned} P_{5.23,1}( \,x, y, t )=\frac{\frac{1}{4} \left( \alpha _2 \mu _3-2 \eta \right) {}^2-\alpha _1^2+4 \alpha _1^4 \mu _2 \left( 3 \text {sech}{}^2\left( -\alpha _1 x-\alpha _2 y+ {}^2\right) -1\right) }{2 \alpha _1^2 \mu _1}. \end{aligned}$$

**Case** (vii): If $\tau _0=\frac{1}{4},~\tau _2=\frac{1}{2} \left( n^2-2\right) ,~\tau _4=\frac{n^4}{4},~ \alpha _1^2\,\mu _1\ne 0,~\text {and} ~0\le n\le 1$, the *JEF* solutions are as follows:

71 $$\begin{aligned} \begin{aligned}&P_{5.24}( \,x, y, t )=-\frac{6 \alpha _1^2 \mu _2\left( \frac{\tau _4 \text {cn}{}^2\left( - \alpha _1 x - \alpha _2 y +\eta t\right) }{\left( \text {dn}\left( -\alpha _1 x - \alpha _2 y+\eta t\right) +\sqrt{1-n^2}\right) {}^2}+\frac{\tau _0 \left( \text {dn}\left( - \alpha _1 x- \alpha _2 y + \eta t\right) +\sqrt{1-n^2}\right) {}^2}{\text {cn}{}^2\left( -\alpha _1 x-\alpha _2 y +\eta t\right) }\right) }{\mu _1}\\&+ \frac{4 \left( -\alpha _2 \eta \mu _3-4 \alpha _1^4 \mu _2 \tau _2-\alpha _1^2+\eta ^2\right) +\alpha _2^2 \mu _3^2}{8 \alpha _1^2 \mu _1}.\\ \end{aligned} \end{aligned}$$

or,

72 $$\begin{aligned} \begin{aligned}&P_{5.25}( \,x, y, t )=\frac{\frac{\left( \alpha _2 \mu _3-2 \eta \right) {}^2}{4} -\alpha _1^2+\alpha _1^4 \mu _2 \left( -\frac{3 \left( (\text {dn}\left( - \alpha _1 x - \alpha _2 y +\eta t\right) +1)^4+\text {sn}^4 \left( - \alpha _1 x - \alpha _2 y +\eta t\right) n^4\right) }{(\text {dn}\left( - \alpha _1 x - \alpha _2 y +\eta t\right) +1)^2 \text {sn}^2\left( - \alpha _1 x - \alpha _2 y +\eta t\right) }-2 n^2+4\right) }{2 \alpha _1^2 \mu _1}.\\ \end{aligned} \end{aligned}$$

A singular periodic wave solutions are obtained by putting $n = 0$ in Eq. (71) and Eq. (72).

73 $$\begin{aligned} P_{5.24,1}( \,x, y, t )=\frac{\left( \alpha _2 \mu _3-2 \eta \right) {}^2-4 \alpha _1^2+16 \alpha _1^4 \mu _2 \left( 1-3 \sec ^2\left( -\alpha _1 x-\alpha _2 y+\eta t\right) \right) }{8 \alpha _1^2 \mu _1}, \end{aligned}$$

74 $$\begin{aligned} P_{5.25,1}( \,x, y, t )= \frac{\frac{1}{4} \left( \alpha _2 \mu _3-2 \eta \right) {}^2-\alpha _1^2+4 \alpha _1^4 \mu _2 \left( 1-3 \csc ^2\left( -\alpha _1 x-\alpha _2 y +\eta t\right) \right) }{2 \alpha _1^2 \mu _1}. \end{aligned}$$

Setting $n = 1$ in Eq. (72) produces a hyperbolic wave solution as follows:

75 $$\begin{aligned} P_{5.25,2}( \,x, y, t )=\frac{\frac{\left( \alpha _2 \mu _3-2 \eta \right) {}^2}{4}-\alpha _1^2-2 \alpha _1^4 \mu _2 \left( \cosh \left( -2 \alpha _1 x-2 \alpha _2 y +2 \eta t\right) +5\right) \text {csch}^2\left( -\alpha _1 x-\alpha _2 y+\eta t\right) }{2 \alpha _1^2 \mu _1}. \end{aligned}$$

All obtained solutions were directly substituted into the governing $(2+1)$-dimensional Wazwaz–Kaur–Boussinesq (WKBE) equation to ensure validity. Through this process, the reduced ODE form (Eq. (7)) together with the auxiliary relation (Eq. (6)) yielded a consistent algebraic system, confirming that the soliton, periodic, exponential, and Jacobi elliptic solutions fully satisfy the original WKBE model.

## Stability analysis of Eq. (1)

The stability analysis of the governing Eq. (1) will be examined in this section. Consider the following disturbed solution^39^:

76 $$\begin{aligned} P(x,y,t)=\gamma \ F(x,y,t)+\phi . \end{aligned}$$

Any constant $\phi$ is a steady-state solution for Eq. (1), as is readily apparent. By entering Eq. (75) into Eq. (1), after using a linearization procedure, we can obtain:

77 $$\begin{aligned} \gamma \ F_{\text {tt}}+\gamma \ \mu _3 \ F_{\text {ty}}-2 \gamma \ \mu _1 \ \phi F_{\text {xx}}-\gamma \ F_{\text {xx}}-\gamma \ \mu _2 \ F_{\text {xxxx}}+\frac{1}{4}\ \gamma \ \mu _3^2 F_{\text {yy}} =0. \end{aligned}$$

Assume Eq. (77) has a solution in the form:

78 $$\begin{aligned} F(x,y,t)=e^{i \left( x \ \mathcal {L}_1+y \ \mathcal {L}_2\right) + \varpi \ t }, \end{aligned}$$

where $\mathcal {L}_1$ and $\mathcal {L}_2$ represent the normalized wave numbers. By inserting Eq. (78) into Eq. (77) and solving for $\varpi$, we obtain:

79 $$\begin{aligned} \varpi \left( \mathcal {L}_1, \mathcal {L}_2\right) =-\frac{1}{2}\ \mu _3\ \mathcal {L}_2 \ i \pm \mathcal {L}_1 \sqrt{-2\ \mu _1 \ \phi +\mu _2 \ \mathcal {L}_1^2-1}. \end{aligned}$$

From Eq. (79), the real part’s sign is always negative for all $\pm \mathcal {L}_1 \sqrt{-2 \mu _1 \phi +\mu _2 \mathcal {L}_1^2-1}<0$, indicating stable dispersion (Fig. 6). The stability criterion depends critically on the expression under the square root in Eq. (79). When $-2\mu _1\phi + \mu _2\mathcal {L}_1^2 - 1 < 0$, the square root becomes imaginary, and the real part of $\varpi$ remains negative, indicating stable behavior. Conversely, when this expression is positive, the stability depends on the specific parameter values and wave numbers.

For clarity, the stability analysis is derived by linearizing the perturbed solution around the exact soliton profile. Substituting the perturbed form into the governing equation and collecting terms of the same order yield the corresponding variational system. The coefficients $\mathcal {L}_1$ and $\mathcal {L}_2$ appear naturally from this process, being determined directly by the model parameters and the soliton solution. Specifically, $\mathcal {L_1}$ measures the influence of dispersive and nonlinear interaction terms, while $\mathcal {L_1}$ arises from higher-order derivative and mixed spatio-temporal contributions. Their relative magnitudes and signs dictate whether perturbations decay or amplify, thus governing the stability of the soliton solutions.

## Graphical representation

**Fig. 1:**
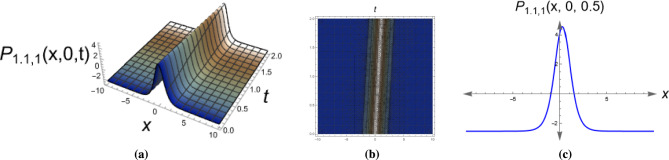
3D, 2D and contour plots of the bright soliton solution for Eq. (9). The figure clearly shows the localized solitary wave with a distinct peak, confirming its bright soliton nature and stability.

**Fig. 2:**
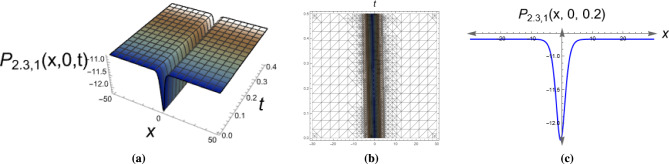
3D, 2D and contour plots of the dark soliton solution for Eq. (16). The plot illustrates a stable intensity dip surrounded by a continuous background, confirming the dark soliton profile observed in optical and fluid systems.

**Fig. 3:**
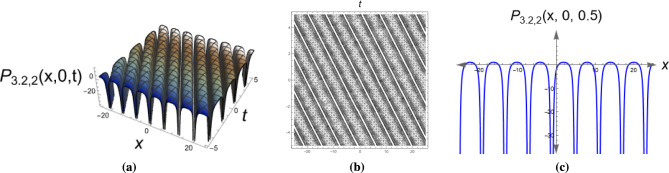
3D, 2D and contour plots of the singular periodic wave solution for Eq. (20). The figure depicts regular oscillatory patterns, verifying the periodic wave structure. Singular features reflect instability at certain parameter regimes.

**Fig. 4:**
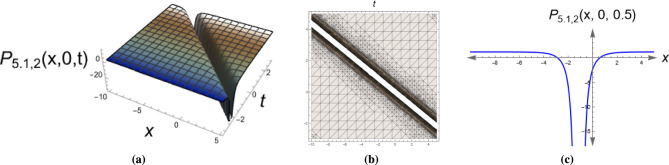
3D, 2D and contour plots of the singular soliton solution for Eq. (30). The plots highlight the blow-up behavior at specific spatial points, a hallmark of singular solitons, representing wave collapse phenomena.

**Fig. 5:**
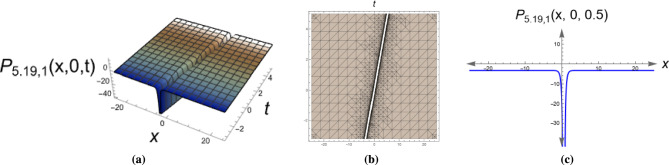
3D, 2D and contour plots of the combo singular-dark soliton solution for Eq. (63). The figure shows both a localized intensity dip (dark soliton) and a blow-up region (singular soliton), confirming the hybrid nature of the solution.

**Fig. 6:**
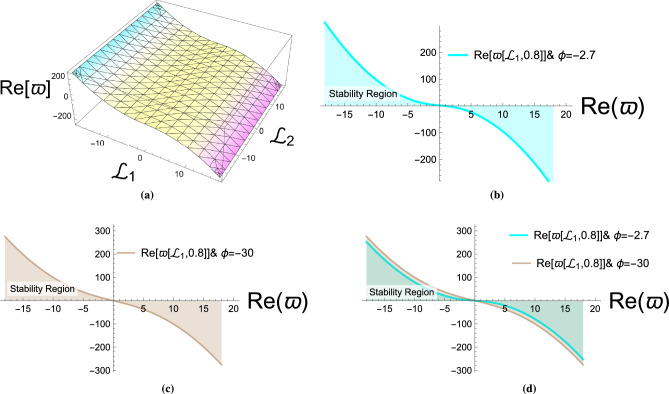
Stability regions obtained from the analysis of Eq. (79). The plots are generated using the parameters $\mu _1 = 0.7$, $\mu _2 = 0.6$, $\mu _3 = -0.7$, $\alpha _1 = 0.8$, $\alpha _2 = 0.5$, and $\eta = 1.0$. The phase parameter is fixed as $\phi =-2.7$ in subfigure (b), $\phi =-30$ in subfigure (c), and a combined representation of both cases is displayed in subfigure (d). The normalized wave numbers are chosen as $\mathcal {L}_1 = 1.0$ and $\mathcal {L}_2 = 0.5$. These diagrams highlight stability zones in the parameter space, confirming the analytical stability analysis presented in Sect.  4.

This section presents the 2D, 3D, and contour plots of selected solutions to illustrate their physical characteristics and dynamical behavior. The figures demonstrate various wave phenomena described by the extracted solutions. Figure 1 displays a bright soliton solution of Eq. (9) with parameters $\alpha _1=0.9, \,\alpha _2=0.95, \,y=0,\,\eta =0.5, \,\mu _1=1.5, \,\mu _2=0.73, \,\mu _3=-0.67, \,\tau _2=1.$ *This solution represents a localized wave pulse that maintains its shape and amplitude during propagation, commonly observed in optical fibers and shallow water waves.* Figure 2 illustrates a dark soliton solution of Eq. (16) with parameters $\alpha _1=1, \,\alpha _2=\sqrt{2},\,y=0, \,\eta =1, \,\mu _1=-2.5, \,\mu _2=0.5, \,\mu _3=\sqrt{2}, \,\tau _2=-0.5.$ *Characterized by a intensity dip on a continuous wave background, dark solitons appear in nonlinear optics and Bose-Einstein condensates.* Figure 3 shows a singular periodic wave solution of Eq. (20) with parameters $\alpha _1=0.62, \,y=0, \,\alpha _2=0.5, \,\eta =-1.6, \,\mu _1=0.8, \,\mu _2=0.73, \,\mu _3=-0.58, \,\tau _2=-0.55.$ *This solution describes periodic wave patterns with singularities, modeling wave breakdown phenomena in fluid dynamics.* Figure 4 presents a singular soliton solution of Eq. (30) with parameters $\alpha _1=0.7, \,\alpha _2=0.59, \, y=0,\,\eta =-1.6, \,\mu _1=0.82, \,\mu _2=0.79, \,\mu _3=-0.4.$ *Singular solitons represent waves with infinite energy concentration at points, useful in modeling rogue waves and extreme events.* Figure 5 demonstrates a combo singular-dark soliton solution of Eq. (63) with parameters $\alpha _1=0.7, \,\alpha _2=0.6, \,y=0,\,\eta =0.75, \,\mu _1=0.82, \,\mu _2=0.79, \,\mu _3=-0.5.$ *This hybrid solution combines characteristics of both singular and dark solitons, representing complex wave interactions in nonlinear media.* The consistent visualization approach (2D, 3D, and contour plots) for all solutions enables clear comparison of their spatial structures and propagation dynamics (Fig. 6).

## Conclusion

This study successfully investigated the (2+1)-dimensional Wazwaz-Kaur-Boussinesq equation by applying the modified extended direct algebraic (MEDA) method. A diverse spectrum of exact solutions was secured, encompassing bright, dark, singular, and combo dark-singular solitons, in addition to hyperbolic, singular periodic, exponential, rational, and Jacobi elliptic function solutions.The primary novelty of this work consisted in the first implementation of the MEDA method to the (2+1)-dimensional Wazwaz-Kaur-Boussinesq equation, which generated previously unreported combo dark-singular soliton solutions and an extensive family of Jacobi elliptic function solutions. A comparative analysis with established results in^38^ confirmed the novelty of the obtained solutions and underscored the efficacy and reliability of the MEDA method for tackling a broad class of nonlinear partial differential equations in mathematical physics. The significance of these findings was substantial, as they provided a comprehensive set of exact analytical solutions that served as valuable benchmarks for validating numerical simulations in complex nonlinear systems. Stability analysis confirmed that the dynamics of the soliton solutions were governed by the specific coefficients within the governing equation. Furthermore, a detailed bifurcation analysis was conducted, revealing that the stability of these solutions was highly sensitive to parameters defining the physical environment, such as wave speed and nonlinearity coefficients.Furthermore, the identification of stable soliton propagation regimes under specific parameter conditions held important implications for practical applications in optical communications and hydrodynamic engineering. The discovery of periodic and quasi-periodic solutions indicated potential stability in specific parameter ranges, thereby enriching the understanding of soliton behavior under diverse conditions. To visualize these findings, various graphical representations, including 3D surface plots and contour density profiles, were generated. These figures effectively illustrated the temporal evolution and spatial structure of the solitons, providing deep insights into the influence of critical parameters on their stability and interaction dynamics. These visualizations not only provided intuitive understanding of complex soliton interactions but also confirmed the method’s capability to capture sophisticated nonlinear phenomena, thereby reinforcing the originality and novelty of the present work. Most significantly, this research established the MEDA method as a powerful unified framework for extracting diverse soliton solutions from higher-dimensional nonlinear systems, thereby paving the way for its future application to other complex models in mathematical physics and engineering. Finally, the correctness of all derived solutions was verified by direct substitution into the governing Wazwaz–Kaur–Boussinesq (WKBE) equation, ensuring their full consistency with the original model. This verification process, performed using symbolic computation tools, guaranteed the mathematical rigor of the solutions. The robust methodology developed in this work can be directly extended to investigate other high-dimensional nonlinear evolution equations encountered in fluid mechanics, plasma physics, and nonlinear optics. Future research directions will focus on adapting the MEDA method to fractional-order models and coupled systems of equations. Additionally, performing comparative studies with high-fidelity numerical simulations and, where feasible, experimental data will be crucial for further validating the physical relevance and predictive power of the obtained analytical solutions.

## Data Availability

The datasets used and/or analyzed during the current study are available from the corresponding author upon reasonable request
